# The War Work of the M.O.H.

**Published:** 1916-12-09

**Authors:** 


### THE WAR WORK OF THE M.O.H.

Amidst the multifarious activities called forth by the Great War that of the Medical Officer of Health throughout the country has received little or no recognition in the general press. Yet when the history of this wonderful time comes to be fully written, we venture to think that very few of its peaceful heroes will merit higher honour than this local government official for the untiring energy and enthusiasm with which he has fulfilled the extraordinary duties and responsibilities that have been cast upon him.

One of the most remarkable features of the war is undoubtedly the freedom from epidemic disease which has so far been enjoyed not only by the armies in the field, but also by the civil population at home. At one time epidemic disease was accepted as the inevitable sequel to a campaign, and with a war of the magnitude predicated by the German threat to civilisation there was reason to fear the worst. That disease has laid so light a hand upon the armed forces abroad must be attributed to the splendid organisation of the R.A.M.C., but for the immunity of both soldiers and civilians in the United Kingdom credit is largely due to the work of the Medical Officers of Health. Quite early in the war, on the initiative of the Local Government Board, an excellent *entente* was established between them and the military authorities. From the outset they have been in consultation and co-operation in regard to the sanitary conditions in the ever-increasing number of areas occupied by large bodies of soldiers. Among other matters they have worked together to provide supplies of pure water and their protection from contamination, to secure satisfactory systems of drainage and for the disposal of garbage and other refuse, and to deal adequately with any outbreaks of infectious disease by means of isolation, hospital treatment, and disinfection.

When we consider the immense number of the troops that have had to be housed, often at short notice, in various parts of the country, it will be realised that the achievement of these objects has meant on the part of Medical Officers of Health an amount of arduous work in addition to their ordinary civil duties which only patriotic enthusiasm could have effectively sustained. With their local knowledge and experience, their assistance to the military authorities in reference to all these matters must have been simply invaluable. At the same time, it should be acknowledged that in the successful accomplishment of this patriotic work the military authorities have doubtless been much aided by the counsel of the medical inspectors especially appointed by the Local Government Board to visit all districts in which troops were encamped or quartered, who thus obtained a special knowledge of varying conditions and the measures most applicable to them. As evidence of the friendly relations everywhere prevailing between the military and civil authorities, it may be remembered that throughout the war period they have interchanged for their mutual information notifications of cases of infectious disease occurring among the military and civil population respectively. It has become the practice, before a new camp is formed or troops billeted in a new district, for the Medical Officer of Health to be informed in order that his expert knowledge may be utilised in the selection of sites for camps or of streets for billeting.

Another important subject in regard to which the assistance of the Medical Officers of Health has been sought and readily obtained is the supply of food to the troops. As contracts with firms were entered into, they were asked through the Local Government Board to undertake the supervision of the factories, etc., concerned, both as regards sanitary conditions and the hygienic qualities of the materials employed. In this work, it should be noted, they had to obtain the co-operation of their sanitary inspectors, to whom a share of the credit for its excellence is due. The food supply thus supervised covered not only the rationing of the troops whilst in this country, but also included all the food materials of home manufacture supplied to the foreign expeditionary forces.

In the early weeks of the war, more especially when Belgian refugees arrived in thousands, Medical Officers of Health had a most onerous duty to perform in examining the newcomers for the purpose of detecting any cases of suspicious illness. It is satisfactory to find in the official records of these and similar matters that recognition is given to these special services. And in the fulness of time doubtless we shall have a complete and detailed appreciation by those best qualified to give it of the war work of the Medical Officer of Health, to become a valued document in the archives of our local government.

